# Structural and Biochemical Characterization of Human PR70 in Isolation and in Complex with the Scaffolding Subunit of Protein Phosphatase 2A

**DOI:** 10.1371/journal.pone.0101846

**Published:** 2014-07-09

**Authors:** Rebecca Dovega, Susan Tsutakawa, Esben M. Quistgaard, Madhanagopal Anandapadamanaban, Christian Löw, Pär Nordlund

**Affiliations:** 1 Department of Medical Biochemistry and Biophysics, Karolinska Institutet, Stockholm, Sweden; 2 Life Science Division, Lawrence Berkeley National Lab (LBNL), Berkeley, California, United States of America; 3 Department of Physics, Chemistry and Biology, Linköping University, Linköping, Sweden; 4 School of Biological Sciences, Nanyang Technological University, Singapore, Singapore; Instituto de Tecnologica Química e Biológica, UNL, Portugal

## Abstract

Protein Phosphatase 2A (PP2A) is a major Ser/Thr phosphatase involved in the regulation of various cellular processes. PP2A assembles into diverse trimeric holoenzymes, which consist of a scaffolding (A) subunit, a catalytic (C) subunit and various regulatory (B) subunits. Here we report a 2.0 Å crystal structure of the free B’’/PR70 subunit and a SAXS model of an A/PR70 complex. The crystal structure of B’’/PR70 reveals a two domain elongated structure with two Ca^2+^ binding EF-hands. Furthermore, we have characterized the interaction of both binding partner and their calcium dependency using biophysical techniques. Ca^2+^ biophysical studies with Circular Dichroism showed that the two EF-hands display different affinities to Ca^2+^. In the absence of the catalytic C-subunit, the scaffolding A-subunit remains highly mobile and flexible even in the presence of the B’’/PR70 subunit as judged by SAXS. Isothermal Titration Calorimetry studies and SAXS data support that PR70 and the A-subunit have high affinity to each other. This study provides additional knowledge about the structural basis for the function of B’’ containing holoenzymes.

## Introduction

Protein phosphatase 2A (PP2A) is a major Ser/Thr protein phosphatase that together with Protein phosphatase 1 accounts for more than 90% of all dephosphorylation events in any given tissue or eukaryotic cell. Reversible phosphorylation and dephosphorylation events are essential for the regulation of a multitude of cellular functions [1], [2]. PP2A participates in various pathways controlling metabolism, DNA replication, transcription, RNA splicing, translation, cell cycle progression, morphogenesis, development and transformation [3]–[6]. To target a broad range of cellular substrates with sufficient specificity, PP2A assembles into diverse trimeric holoenzymes.

Each holoenzyme consists of a common core formed by the scaffolding (A) and the catalytic (C) subunit (two isoforms each) and associates with a variable regulatory B-subunit into a heterotrimeric complex [7]–[9]. Four families of regulatory B-subunits, with no homology between them and very different expression levels in different cell types and tissues have been identified to date: B/B55/PR55, B’/B56/PR61, B’’/PR72/PR70 and B’’’/Striatin/PR93 [10], [11]. Within the holoenzyme, the regulatory B-subunits control the function of PP2A by mediating substrate specificity and modulating the catalytic activity. Crystal structures of PP2A holoenzymes with members of the regulatory B, B’ and very recently B’’ subunit families have been determined [12]–[16], shed light on the overall enzyme architecture and given initial ideas on how regulatory subunits might affect and influence PP2A activity.

The scaffolding subunit A (isoforms α and β) consists of 15 tandem huntingtin-elongation-A subunit TOR (HEAT) repeats which form an overall U-shaped conformation [17], [18]. Each HEAT repeat is formed by two antiparallel α-helices, with highly conserved interhelical loops. Structural studies have revealed that HEAT repeats 11–15 interact tightly with the catalytic subunit [12] while the N-terminal HEAT repeats 1–10 bind the diverse set of regulatory subunits [13]–[16], . From the crystal structures of the isolated A subunit [17], the core dimer [12] and the various trimeric holoenzymes [13]–[16] together with molecular dynamics studies [21] it has emerged that the A-subunit is a highly flexible module, which can adapt its shape, i.e. its overall curvature, to the various binding partners.

The structures of known members of the different regulatory B-subunits are highly diverse in terms of sequence and structure. Members of the B’’/PR72/PR70 subunits are encoded by three different genes (see table 1 and reference [9] for nomenclature) and are involved in various cellular processes such as Wnt signaling [22], [23], DNA synthesis, neuronal signaling [24], tumor suppression [3] and cell cycle progression [25]–[27]. For the latter, PR72 members are proposed to play a role in the regulation of the retinoblastoma and cell division control 6 (Cdc6) proteins, which are key proteins for the G1/S transition. Unique for this B-subunit family is the conservation of two EF-hand calcium binding motifs, which are important for the function of the protein. It has thus been shown that calcium binding enhances binding of the PR72 subunit to the core enzyme and significantly affects the phosphatase activity [24], [25], [27], [28].

**Table 1:** PP2A B’’-subunits and their tissue distribution.

Gene	Protein	Aliases	Tissue distribution
*PPP2R3A*	B’’α1	PR130	widespread
*PPP2R3A*	B’’α2	PR72	Heart, skeletal muscle
*PPP2R3B*	B’’β1	PR70 (PR48)	unknown
*PPP2R3B*	B’’β2	PR70	Heart, skeletal muscle
*PPP2R3C*	B’’γ	G5PR	widespread

Here we report the crystal structure of an isolated member of the B’’-subunit, PR70, at 2.0 Å resolution and present a model of the PR70/A-subunit complex based on SAXS data. Furthermore, we have characterized the interaction of both binding partner and their calcium dependency using biophysical techniques. The presented study provides additional knowledge about the structural basis for the function of B’’ containing holoenzymes.

## Results and Discussion

### Construct design and crystallization

**Figure 1:**
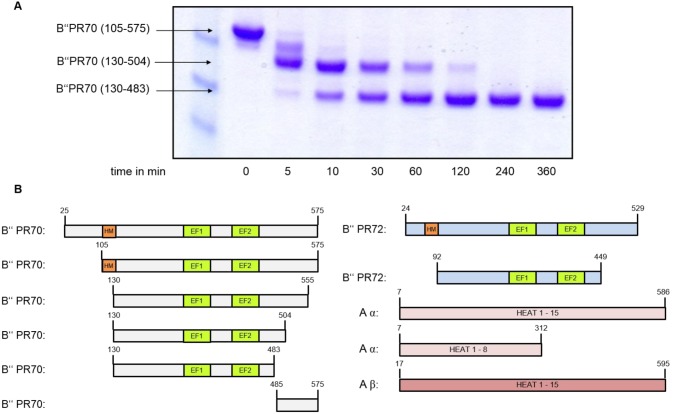
Identification of constructs suitable for X-ray structure determination. **A)** Time course of a limited proteolysis study of a PR70 constructs in the presence of chymotrypsin resulted in a stable core fragment suitable for crystallization. Domain boundaries were determined via mass spectrometry. **B)** Illustration of the functional domains of the B″ subunit (EF-hands and hydrophobic motif) and the various constructs used in this study.

Various constructs of the different members of the B’’-subunit family were designed according to the multi construct approach [29], [30] based on rational design strategies using secondary structure and disorder prediction programs. More than 30 constructs were recombinantly expressed in soluble form, purified and screened for crystallization. None of these proteins gave rise to diffracting crystals. As an alternative approach, we identified a chymotrypsin resistant core of the PR70 subunit (residues 130–483) using limited proteolysis together with mass spectrometry (Figure 1A). A similar stable fragment was also identified for the homologous PR72 subunit (residues 92–449) using the same approach, but only the PR70 core fragment readily crystallized.

### Structure of the PR70 core fragment

**Figure 2:**
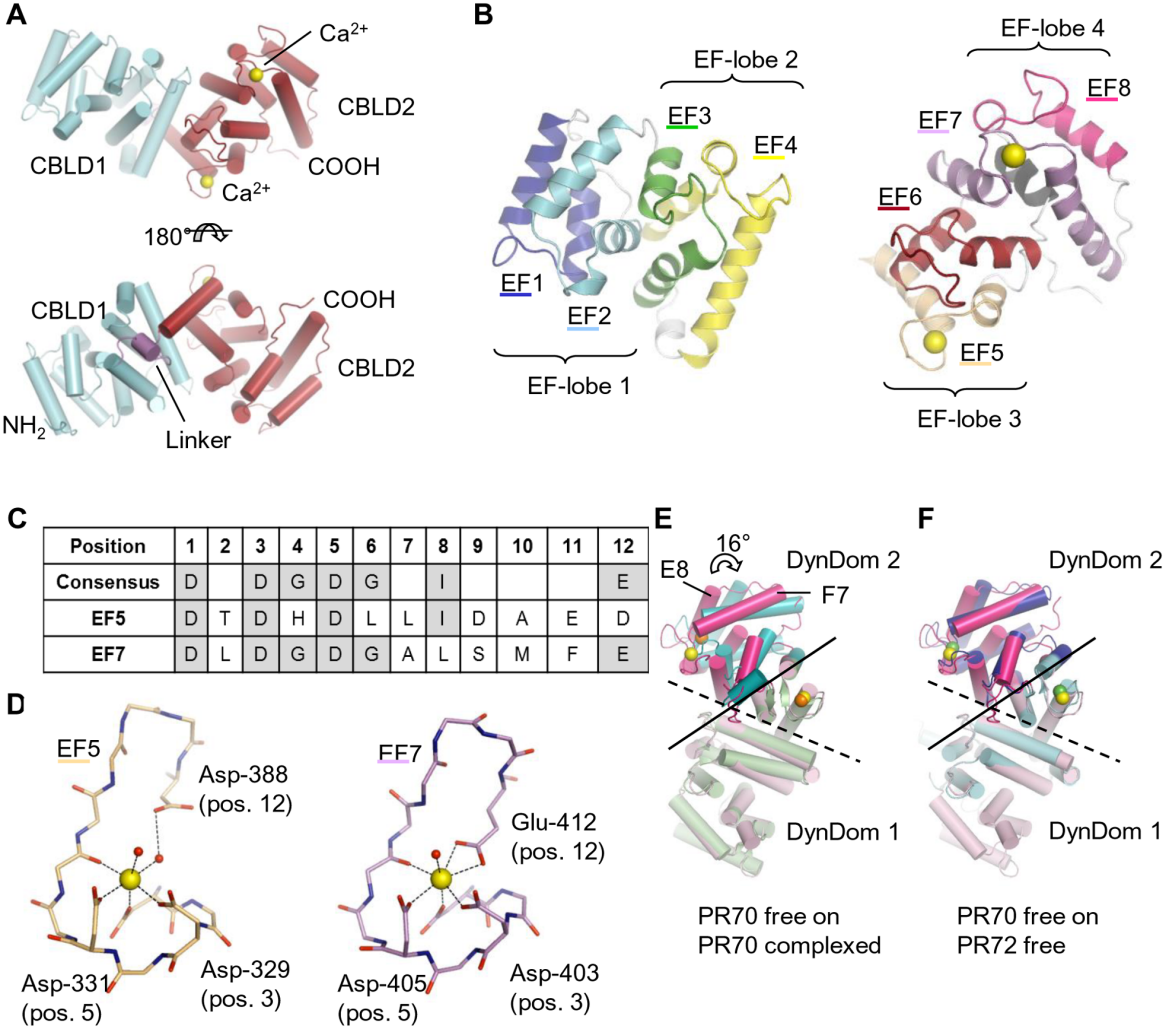
Overall structure of PR70. **A)** Overview of the structure. CBLD1 and 2 are light blue and dark red respectively, the linker between them is violet and calcium ions are yellow. **B)** Composition of the two calcineurin B-like domains. CBLD1 (left) and CBLD2 (right) are both shown in same orientation as in A, top panel. EF hands are blue (EF1), light blue (EF2), green (EF3), yellow (EF4), pale orange (EF5), dark red (EF6), violet (EF7) and pink (EF8), termini and connecting loops are grey, the C-terminal helix is black, and calcium is yellow. **C)** Sequence of EF loops 5 and 7. EF loop consensus sequence is shown. Residues conforming to the consensus are highlighted in grey. **D)** Structure of EF loops 5 and 7. Colored as in B. Water molecules interacting with calcium are red. Side chains are omitted for residues not directly involved in calcium binding (except for Asp-338). **E)** Structural changes upon complex formation. DynDom suggests the presence of two domains that move relative to each other upon formation of the PP2A holoenzyme. Free and complexed PR70 were superimposed using DynDom domain 1. The stippled line marks the boundary between CBLD1 and 2, and the full line marks the approximate boundary between the DynDom domains. For free PR70, DynDom domain 1 and 2 are pale pink and pink respectively and calcium is yellow. For complexed PR70, DynDom domains 1 and 2 are pale green and turquoise respectively and calcium is orange. **F)** Structural comparison of free PR70 and free PR72. Orientation as in E. For PR72, DynDom domains 1 and 2 are light blue and blue respectively and calcium is green.

The structure of the PR70 core fragment (residues 130–483) was solved by selenomethionine single anomalous dispersion (SAD) phasing and refined at 2 Å resolution yielding a final R_free_ of 19.3% (R_work_ = 16.2%) (Table 2). The model encompass residues 134–481. Very recently, the structure of the PR70 core (residues 122–490) was also determined in context of the trimeric PP2A holoenzyme complex [15]. In that study, the PR70 core fragment has been described as an elongated protein with two EF-hands [15]. However, we find that the structure can be more accurately described as a tandem of two calcineurin B-like domains, which we have named CBLD1 and CBLD2, connected by a 12-residue linker (residues 301–312) (Figure 2A). Calcineurin B-like domains contain four EF-hands, which are arranged into two so called EF-lobes, i.e. pairs of tightly interacting EF-hands. Eight EF hands are thus present in the PR70 core fragment; EF1–4 (CBLD1) and EF5–8 (CBLD2) (Figure 2B). In addition to these EF hands, an extra helix is found C-terminally of EF8 following a short turn (Figure 2B). A DALI search for structural homologues of CBLD1 and CBLD2 returned a number of hits with Z-scores >8, which are all calcineurin-B like proteins (Table 3, Figure S2). Notably, the sequence similarities of PR70 with these hits are very low (Table 3), which explains why PR70, to our knowledge, has not previously been predicted to contain calcineurin B-like domains. When using the separated CBLD domains as search models, CBLD2 resulted in smaller RMSD values compared to CBLD1 (Table 3), suggesting that CBLD1 is a rather atypical calcineurin B-like domain. This seems to be partly due to an unusual large twist angle between EF-lobes 1 and 2. Indeed, repeating the DALI search with the individual EF-lobes resulted in drastically improved RMSD values. Furthermore, the divergence between values for EF-lobes 1 and 2 compared to EF-lobes 3 and 4 is much smaller than between CBLD1 and CBLD2 (Table 3).

**Table 2:** Data collection, phasing and refinements statistics.

	PR70 core fragment
**Data collection**	
Beamline	DLS I04
Wavelength (Å)	0.9796
Space group	P2_1_
Cell dimensions	
*a*, *b*, *c* (Å)	37.11, 74.46, 62.33
α, β, γ (°)	90, 92.63, 90
Resolution (Å)	37.23–1.99 (2.04–1.99)
*R*_sym_ (%)	9.7 (60.1)
*I/*σ*I*	13.8 (2.4)
Completeness (%)	99.1 (89.0)
Redundancy	3.8 (3.2)
**SAD phasing**	
No. selenium sites	10
Figure of merit	0.45
**Refinement**	
No. reflections	45105
*R*_work_/*R*_free_ (%)	16.2/19.3
No. atoms	
Protein	2844
Calcium	2
Solvent (glycerol)	12
Solvent (water)	347
*B*-factors	
Protein	22.8
Calcium	18.9
Solvent	32.7
R.m.s. deviations	
Bond lengths (Å)	0.004
Bond angles (°)	0.803
Ramachandran plot	
Favored (%)	98.6
Outliers (%)	0

Numbers in parentheses indicate statistics for the highest resolution shell.

**Table 3:** DALI search results: Structural relatives of the CBLD domains and individual EF-lobes of PR70.

PR70	Hits	Z-score	RMSD(Å)	Aligned residues	% Seq. identity
CBLD1	KChIP1 (human)	9.3	4.8	141	16
	UNP: Q9NZI2, PDB: 1S1E				
	NCS-1 (human)	8.5	5.8	138	11
	UNP: P62166, PDB: 1G8I				
	Calcineurin B (human)	8.4	4.6	126	10
	UNP: P63098, PDB: 2P6B				
	Frequenin (S. cerevisiae)	8.3	6.3	137	15
	UNP: P39104, PDB: 2JU0				
	CBL4 (A. thaliana)	8.2	4.5	117	10
	UNP: O81223, PDB: 1V1F				
CBLD2	CBL2 (A. thaliana)	11.1	3.3	146	14
	UNP: Q8LAS7, PDB: 1UHN				
	Neurocalcin-δ (B. Taurus)	11.0	3.3	150	15
	UNP: P61602, PDB: 1BJF				
	CBL4 (A. thaliana)	10.7	3.9	147	19
	UNP: O81223, PDB: 1V1F				
	KChIP1 (human)	10.6	3.3	145	10
	UNP: Q9NZI2, PDB: 1S1E				
	Calcineurin B (human)	10.3	3.9	135	14
	UNP: P63098, PDB: 2P6B				
EF-lobe 1	Calmodulin N-term (human)	8.0	2.4	66	14
	UNP: P62158, PDB: 3UCY				
EF-lobe 2	Calcium-binding p22 (rat)	8.3	2.5	71	8
	UNP: P61023, PDB: 2CT9				
EF-lobe 3	CDC31p (S. cerevisiae)	9.0	1.8	65	20
	UNP: P06704, PDB: 2DOQ				
EF-lobe 4	KChIP1 (human)	8.7	2.2	72	11
	UNP: Q9NZI2, PDB: 2I2R				

For CBLD1 and 2, the five top scoring hits (highest Z-scores) are listed, while only the best match is listed for EF-lobe 1–4. Hits are listed along with their UniProt (UNP) and PDB accession codes, Z-score, RMSD value, number of aligned residues and percent sequence identity. Structural overlays for the best DALI matches are shown in figure S2.

The EF-hand is a helix-loop-helix motif (E-helix, EF-loop, F-helix), which often has the capacity to bind calcium. In a canonical EF-hand, the EF-loop is twelve residues in length and contains aspartic residues in positions 1, 3, 5, glycines in positions 4, 6, and a glutamate in position 12, which may in rare cases be substituted by an aspartate (Figure 2C) [31], [32]. In agreement with previous observations, only two of the EF hands of PR70 are loaded with calcium [15], [25], [27]. These EF hands correspond to EF5 and EF7 in the present structure, but have in the previous literature been referred to as EF1 and EF2 respectively (Figure 1, 2). EF7 is a canonical EF hand (Figure 2C). In agreement with this, the coordination number for the chelated Ca^2+^ ion is seven. Just one of the interactions is contributed by a water molecule, and the coordination geometry is pentagonal bipyrimidal, which is the preferred coordination geometry for calcium [31] (Figure 2D).

On the other hand, EF5 is distinctly non-canonical: it lacks the two conserved glycine residues, and position twelve is occupied by an aspartate (Figure 2C, D). Despite these sequence deviations, the loop geometry conforms fairly well to a typical EF-loop. Substitution of the canonical glutamate for an aspartate in position 12 usually leads to a more compact EF-hand, as the shorter side chain has to reach further to interact with the Ca^2+^ ion [32]–[34]. However, in EF5, the aspartate does not interact directly with the Ca^2+^ ion, but forms a single indirect interaction mediated by a water molecule instead. This results in a wider loop structure with a larger angle between helices E5 and F5 (∼117°) as compared to E7 and F7 (∼91°). As a result of this unusual arrangement, the chelated Ca^2+^ ion is coordinated by only six atoms in an octahedral pattern, with two of them being water molecules (Figure 2D). The structure thus agrees well with the suggestions that EF5 binds calcium with lower affinity than EF7 [15], [27]. All other EF-hands of PR70 deviate markedly from the consensus sequence. Although an acidic amino acid is always found in or close to position 12, and one of the consensus glycine residues is usually also present, aspartic acids present in positions 1, 3 and 5 of a canonical EF-hand are consistently missing. Therefore it is not surprising that none of these EF-hands were found to bind calcium. In terms of structure, EF-loop 4 displays fairly typical EF-loop geometry, while EF-loops 3, 6 and 8 only show typical geometry for positions 6–12. EF-loops 1 and 2 are both highly atypical.

A comparison of the crystal structures of PR70 in isolation and in complex with the catalytic and scaffolding subunit suggests considerable conformational changes upon complex formation (Figure 2E, F). This is likely not caused by rigid body movements of CBLD1 and CBLD2 relative to each other, as helix E4 from CBLD1 interacts with the inter-domain linker as well as helices E5 and F6 from CBLD2 forming an extensive and probably rather rigid hydrophobic interface (Figure S3). Using the program DynDom, we have identified two putative rigid body domains, which move relative to each other by a ∼16° rotation upon complex formation; 1) CBLD1 and most of EF5 and helix F6 (residues 137–339 and 371–381), and 2) the remainder of CBLD2 (Figure 2E). It should be mentioned that the conformation observed for free PR70 might to some extent be caused by constraints imposed by crystal packing. However, the conformation of free PR70 is considerably more similar to that of homologous free PR72 than to complexed PR70 [15], suggesting that the observed structure is typical for free PR70/72 (Figure 2F). Specifically, the RMSD Cα is 0.9–1.0 Å for free PR70 superimposed on free PR72 while it is 1.7–1.9 Å for free PR70 on the complexed form of PR70. However, when performing the superimpositions using only DynDom domain 1, much lower values are obtained; 0.6–0.7 Å for free PR70 on free PR72 and 0.6 Å for free PR70 on complexed PR70, as would be expected if a structural change occurs between DynDom domains 1 and 2 upon complex formation. Although the structural differences between the free and bound form of PR70 are rather modest, they are likely to be important, as superimposing free PR70 on complexed PR70 using DynDom domain 1, results in numerous major clashes with the PP2A scaffolding subunit as well as in a loss of interactions with the catalytic subunit. We therefore propose that modest but relevant structural conformational changes occur in the PR70 core domains upon PP2A holoenzyme assembly in unison with the conformational change previously described for the PP2A scaffolding A subunit [15], [35].

### Thermal Stability of PR70 is Ca^2+^ dependent

**Figure 3:**
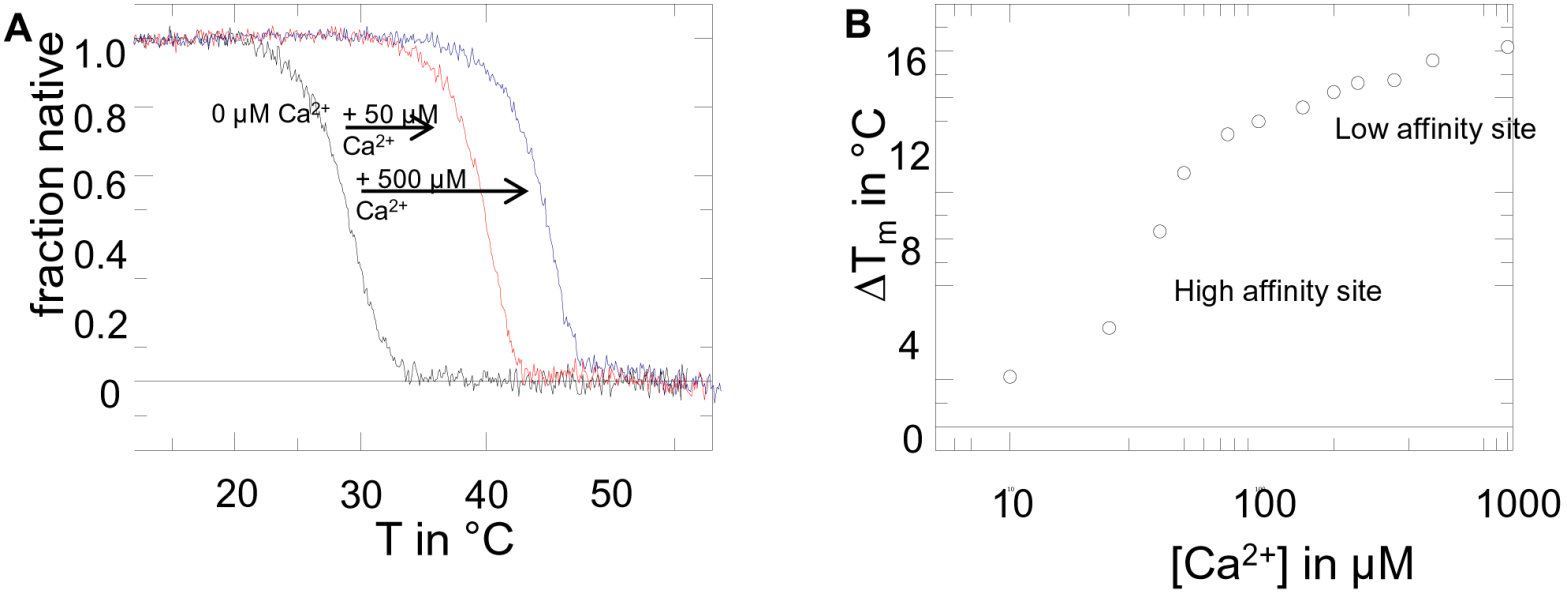
Calcium dependent thermal stability of PR70. **A)** Representative thermal unfolding curves of the PR70 construct (residues 105–575) are shown in the presence of the different Ca^2+^ concentrations. **B)** Ca^2+^-dependence of thermal unfolding suggests a low and high affinity Ca^2+^ binding site.

Calcium regulation has been previously described as a unique regulatory feature for members of the B’’-subunit family. Crystal structures of PR70 and PR72 in isolation or in complex with other PP2A subunits confirmed the binding capacity of up to two Ca^2+^ ions *via* EF-hand motifs. To unravel the role of calcium further, we measured the stability of a couple of PR70 constructs and did a thermal unfolding measurment in the presence of different concentrations of calcium on one (residues 105–575). To deplete calcium to a high extent, the protein was initially purified without the addition of extra calcium and further dialyzed against EGTA containing buffer. Ca^2+^-free PR70 preparations were very prone to aggregation and displayed an unfolding temperature below 30°C (Figure 3A). Upon stepwise addition of calcium (up to 1 mM), the protein was significantly stabilized upon heat denaturation with an unfolding midpoint 16°C higher at saturation than the Ca^2+^-free protein. Analysis of unfolding temperatures versus calcium concentration (Figure 3B), suggests two calcium binding events, representing the low and high affinity Ca^2+^-binding sites respectively. Interestingly, the PR70 protein purified without additional calcium (but not treated with EGTA) displays a stability of around 40°C, indicating that calcium is tightly bound in the high affinity binding site, while higher calcium concentrations are necessary to occupy the low affinity site. We therefore propose that calcium in the high affinity binding site ( = EF7) maintains the structure and stability of the protein and ensures the integrity of the interaction surface with the scaffolding A subunit in the trimeric complex, even at low ground-state calcium concentrations. On the other hand, the second, quite unusual low affinity calcium binding site might play a role in the regulation of PP2A binding to its substrates and to the activity as proposed earlier [15], in response to changes in calcium concentration upon cell signaling.

### A- and B’’-subunit interact with high affinity

**Figure 4:**
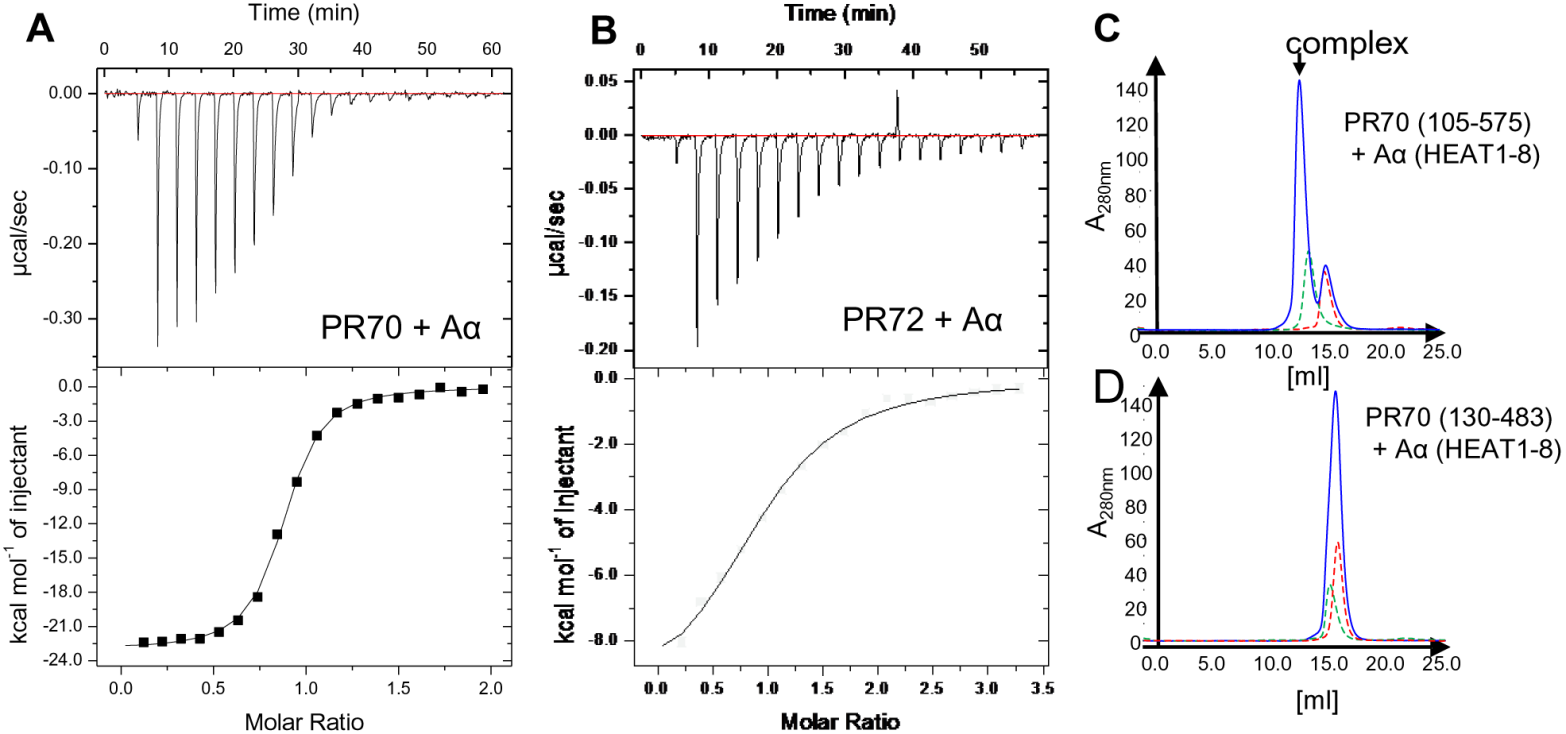
Interaction of A and B’’-subunits. **A)** Isothermal titration of the PR70 subunit (residues 25–575) with the full length A-subunit. Raw data and integrated heats are shown. **B)** Same as in A), but here the PR72 subunit (residue 24–529) was titrated with the A-subunit. Resulting thermodynamic parameters are shown in Table 3. **C,D)** Gel filtration of individual (green and red) and mixed (blue) A- and B subunits to monitor complex formation. The longer PR70 construct (residue 105–575) is able to form a stable complex on gel filtration while the core PR70 fragment (130–483) has lost its ability for tight interaction.

In an alternative approach to optimizing the crystallization properties of the PR70 subunit, we screened for binding partners to PR70 to reduce its flexibility and increase the chance for crystal formation. By using analytical gel filtration and isothermal titration calorimetry (Figure 4), we surprisingly found that the scaffolding A subunit forms a tight complex (K_D_ ≈50 nM to 3 µM; depending on the B’’-subunit) with members of the B’’-subunit family *in vitro* even in the absence of the catalytic C subunit. We could not show that any of the well characterized members of the B- and B’-subunits interact with high affinity with the scaffolding subunit only either on gel filtration or ITC (data not shown). To characterize the interaction between the B’’-subunit members and the scaffolding A subunit further, we made use of the various B’’-constructs used for crystallization (Figure 1B) and tried to identify the minimal core constructs that maintained high affinity binding. Initially we generated a deletion series of the scaffolding A subunit by successively deleting one HEAT repeat at a time starting from the C-terminus. ITC data revealed that a construct spanning HEAT repeats 1–8 is sufficient for high affinity binding of PR70, which is in good agreement with the recently published trimeric holoenzyme structure (Table 4). Interestingly the affinity of the β-isoform of the scaffolding A subunit to PR70 constructs is 5–10 fold reduced compared to the α-isoform, albeit these proteins share more than 90% sequence identity. This was also shown earlier for the other isoform PR72 [36]. Residues of the A subunit of both isoforms involved in the interaction with the PR70 subunit are highly conserved and do not explain the observed affinity difference. Despite their high sequence identity both A subunit isoforms differ significantly in their stability against heat denaturation (more than 15 degrees; unpublished results), which could be the reason for the differences in binding affinity.

**Table 4:** ITC derived binding affinities of B’’-constructs to A-subunits.

B’’-subunit (residues)	A-subunit	N^a^	K_D_ (µM)	ΔH (kcal/mol)
PR72 (24–529)	A α (HEAT1–15)	0.94±0.01	2.92±0.30	−10.2±0.5
PR72 (92–449)	A α (HEAT1–15)		NB	
PR70 (25–575)	A α (HEAT1–15)	0.84±0.02	0.112±0.006	−22.6±0.1
PR70 (105–575)	A α (HEAT1–15)	0.85±0.03	0.045±0.008	−19.7±0.2
PR70 (130–555)	A α (HEAT1–15)		>100	
PR70 (130–504)	A α (HEAT1–15)		>100	
PR70 (130–483)	A α (HEAT1–15)		>100	
PR70 (485–575)	A α (HEAT1–15)		NB	
PR70 (25–575)	A β (HEAT1–15)	1.01±0.02	0.702±0.081	−21.5±0.4
PR70 (105–575)	A β (HEAT1–15)	0.86±0.03	0.411±0.043	−17.1±0.2
PR70 (25–575)	A α (HEAT1–8)	0.93±0.02	0.223±0.021	−19.1±0.4
PR70 (105–575)	A α (HEAT1–8)	0.99±0.01	0.158±0.022	−20.3±0.2
PR70 (130–504)	A α (HEAT1–8)		>100	
PR70 (130–483)	A α (HEAT1–8)		>100	

ITC, isothermal titration calorimetry; NB, no binding detected.

a N = stoichiometry (A-subunit to B-subunit).

In addition, although the B’’-subunits PR70 and PR72 share a similar core with high sequence identity (Figure S1), their affinities towards binding of the A-subunit differ 30-fold. While most residues directly involved in binding of the A-subunit according to the recently determined complex structure are conserved in both isoforms (except for R288 in PR70, which is a K in PR72, Figure S1), their sequences mainly differ in the N-terminal region.

Table 4 summarizes the binding data obtained via ITC for the different A- and B’’-constructs. This shows that the PR70 core fragment (residues 130–483) used for structure determination is not sufficient for high affinity binding to the A-subunit. Affinity loss is not caused by the deletion of the C-terminal 91 residues, which are predicted to be in a random coil conformation and serve as a binding platform for PP2A substrates [26], [27]. A patch outside the identified core fragment spanning residues 105 to 129 significantly contributes to binding. In the recent holoenzyme structure residues 125–129 were found to mediate a number of additional hydrophobic interactions with the A-subunit and were referred to as the ‘hydrophobic motif’.

We also studied the influence of calcium on the binding affinity between the PR70 construct and the A-subunit. Protein prepared with a calcium-free buffer (but not treated with EGTA) bound the A-subunit with identical affinity compared to the conditions with excess of calcium. No binding event could be detected when the samples were treated with EGTA to chelate out all calcium ions (data not shown). However, it should be noted, that Ca^2+^-free PR70 was highly prone to aggregation.

### Structural model of the PR70/A-subunit complex – SAXS

**Figure 5:**
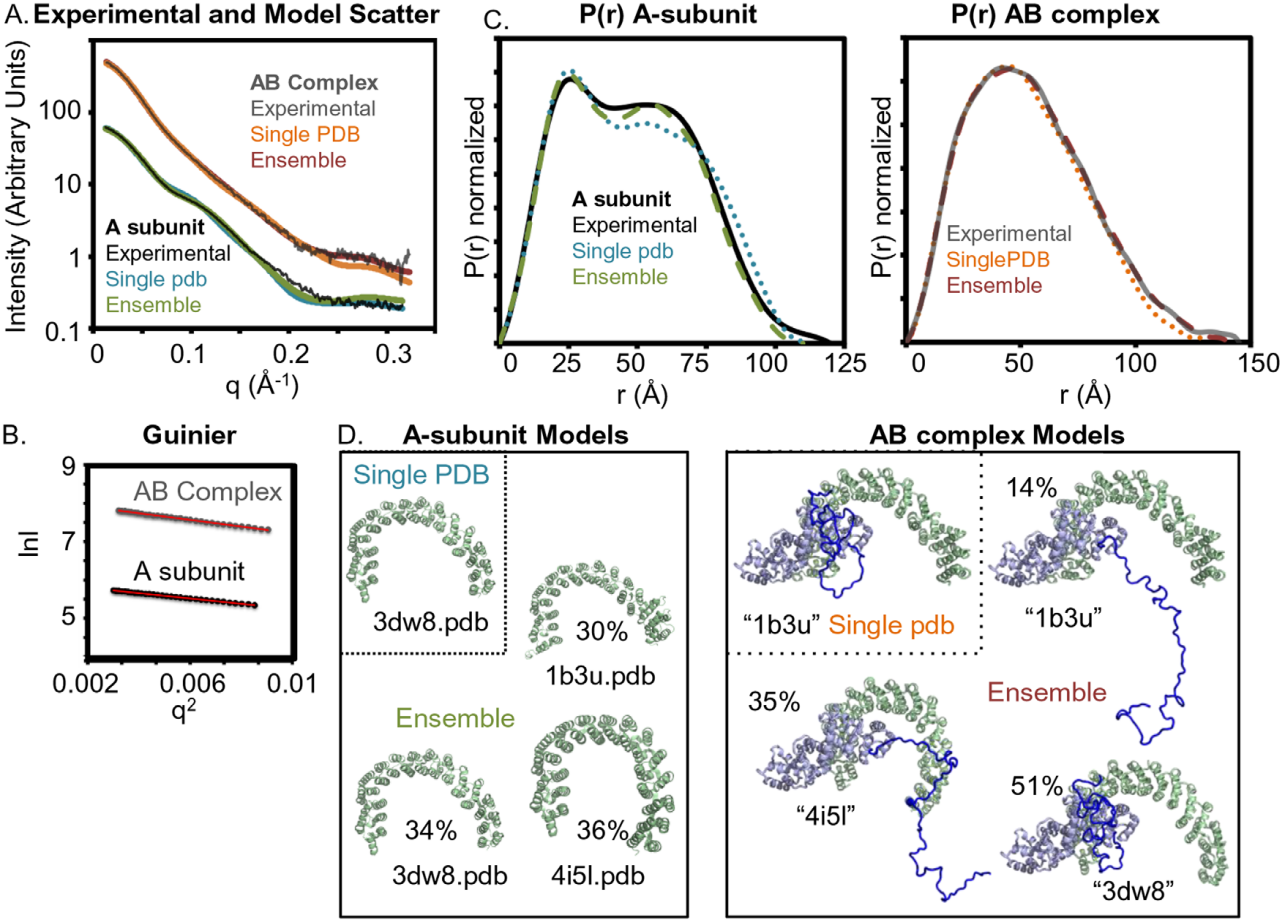
SAXS analysis of A-subunit and AB’’ complex suggests that the A-subunit is highly flexible. Single models are generally consistent with the overall experimental scatter and p(r) distribution. However, for both the A-subunit and the AB’’ complex, an ensemble of models with A subunits with different conformations better fit the experimental data. **A)** SAXS plots of the experimental A-subunit and the AB’’ complex data overlaid with predicted scattering curves from the models that best fit the experimental scattering data. Predicted SAXS curves of pdb models or ensembles of PDB models were generated using FOXS. **B)** The linearity of the Guinier plots are consistent with non-aggregated samples. **C)** P(r) plots of experimental data and of single pdb models or an ensemble of PDB models. **D)** Cartoon depictions of A-subunit models (green) and AB’’ complex models (green/blue) most consistent with the experimental SAXS data. A-subunit models were taken from representative pdbs. AB’’ complex models (blue) were based on the published complex structure 4i5l with the A-subunit of 4i5l being replaced with A-subunits from other pdbs. The C-terminus (dark blue) was modeled using BilboMD.

To complement our crystal structure of the PR70 subunit, we used Small Angle X-ray Scattering (SAXS) to determine the structure of the A- and PR70 complexes in solution (Figure 5). Proteins were purified by size exclusion chromatography (SEC) just prior to SAXS data collection, and resulting Guinier plots were linear, consistent with monodisperse protein preparations. The A-subunit molecular mass calculated from the SAXS was 62.5 kD, within error of the calculated molecular weight of 65.3 kD.

Comparison of the A-subunit experimental SAXS data with scattering curves predicted from 4 A-subunit atomic models derived from representative crystal structures were consistent with each other but showed significant differences (Figure 5). The Chi free error of the fit from comparing the individual models to the data ranged from 9 to 15, with the best fit to an A-subunit only crystal structure (1b37.pdb) and the worst fit being the A subunit from the trimeric PP2A holoenzyme complex [15]. Given the fact that the 15 HEAT repeats of the A-subunit are in multiple conformations in different crystal structures, we examined the fit of an ensemble of A-subunits to the experimental data in solution. We observed a significant improvement of the fit with a Chi free error reduced to 4.3. This ensemble was 30% to the heat domain from 1b3u.pdb, 34% to that from 3dw8.pdb (holoenzyme with B55), and 36% to that from 4i5l.pdb(holoenzyme with PR70). Although the ensemble did show an improved fit to the data based on the Chi free error, there were still significant differences – implying that the A-subunit models derived from different crystal structures do not reflect the conformations of the A-subunit in solution. It has been noted previously, that the most significant differences between various A-subunit models occur between HEAT repeat 10 and 11. Therefore, we compared the HEAT-to-HEAT repeat distances in the four representative structures, as defined by measuring the distance from the middle of each heat domain, as the ends might vary from heat domain to heat domain. In particular, we looked at distances between the Cα atoms of A-subunit residues 13, 52, 90, 129, 167, 206, 245, 284, 327, 366, 405, 445, 483, 522, and 561. Although the largest conformational change occurred between HEAT repeat 10 and 11 (4.1 Å), there were additional significant changes in the HEAT-to-HEAT repeat distance of 0.5 to 1.2 Å whose movements together likely contribute differing conformations in solution, as previously suggested in the MD studies [21]. It is likely that an ensemble of the MD generated conformations will better fit the experimental scattering curve than the crystal structures whose conformations may be limited by crystal contacts, as we previously observed with ubiquitinated PCNA [37].

To analyze the SAXS data of the complex between the A- and PR70 (construct 130–575) subunits, we needed to make a model, as a structure of the construct 130–575 has not been determined. The published crystal structure of the trimeric PP2A with the PR70 subunit contained the respective A-subunit, but went from 120–478 in the PR70 subunit [15]. Therefore, we took the A and PR70 subunits from the crystal structure (4i5l.pdb), removing the PR70 subunit N-terminal ten residues and modeled in additional residues to correlate to our solution data with the PR70 subunit from 130–575. We then used BilboMD to create atomic models to fit the experimental scattering data. BilboMD uses the molecular dynamics CHARMM program to create atomic models, where structured regions can be maintained as rigid bodies and flexible loops and termini are “allowed to move” in the molecular dynamics program [38]. Bond lengths are kept constant. Using a minimal ensemble search, the BilboMD program will identify ensembles (2–5 models) that together will be similar to the scattering curve. The program will also calculate the fit of every single individual model to the experimental scattering program, identifying the model that best fits the experimental data, based on Chi. This program thus is useful in obtaining atomic models that are consistent with the experimental SAXS data and in the ensemble analysis, identifying the flexibility that might be occurring in solution. In this study, residues 130–478 of PR70 modeled in the crystal structure and the A-subunit were kept as a rigid body. The C-terminus, which we postulated to be flexible, was allowed to move in the molecular dynamics program. We first ran BilboMD based on the complex model (4i5l), as described above. Based on our experimental SAXS results of the A subunit in solution being flexible, we also generated models where the A subunit was replaced with A subunits from other A subunit crystal structures. Comparing the ensembles chosen from the different BilboMD runs, the Chi free error for the fit of the experimental data to an ensemble of complex models was 2.7 (based on heat domain from 4i5l.pdb), 0.93 (based on heat domain from 3dw8.pdb); 1.3 (based on heat domain from 2npp.pdb); and 1.1 (based on heat domain from 1b37.pdb). A significant population of selected models had a collapsed PR70 C-terminal region, suggestive of a folded region. Combining all the models from the different BilboMD runs and using a MES search to the experimental scattering data identified a group of three structures that gives a Chi free error of 0.92. The best ensemble with relative population percentages were 51% model derived with A subunit from 3dw8.pdb, 14% model derived from 1b37, and 35% from model derived from 4i5l. Notably, there is not a strong difference in the Chi fit between the MES search based on the BilboMD run with 3dw8 and the MES search with the mixed populations. Therefore, to obtain an complementary measure, we used a Volatility Ratio (V_R_) measure [39]. V_R_ is much less sensitive to *R_g_* than Chi free and can be used as an orthogonal measure for how well the model matches the experimental data. The V_R_ Similarity to the experimental data were 0.70 and 0.30 for the 3dw8 BilboMD run and for the mixed population MES, respectively. As 0 is expected for a perfect fit, the V_R_ measure suggests that the MES with the mixed population better agrees with the experimental data. This MES analysis suggests that in the complex between the A- and PR70 subunits, the heat 15 domains are still mobile and that the PR70 is flexible but may be partially folded.

## Conclusion

In this work we complement the very recent structure of PR70 in the heterotrimeric PP2A complex with a high resolution structure of the free PR70, as well as biochemical and low resolution data describing it’s interactions with the A-subunit in a dimeric complex. The structure of PR70 in the heterotrimer shows small but significant structural differences as compared with free PR70, supporting a degree of induced fit upon binding. Our biophysical studies emphasize a role for calcium in regulating the stability of PR70 and in the interaction to the A-subunit, in agreement with previous studies [24], [25], [27]. Interesting, we show that PR70 and the A-subunit have high affinity to each other, opening for a scenario where this complex can be formed before the C-subunit is recruited to the heterotrimer. This is in contrast to the assembly of heterotrimers containing B- and B’-subunits where the initial formation of the core dimer is necessary [40].

Although we failed in crystallizing the PR70-A-subunit heterodimer we used SAXS to study the complex in solution. Overall, the SAXS data of the A and PR70 subunit complex is consistent with the crystallographic structure of the entire complex including the catalytic domain. However, there are obvious differences, primarily with regard to the A subunit. SAXS data of the A subunit only were consistent with heat domains shifting relative to each other. The flexibility of the A-subunit has general functional relevance, as flexibility will both allow interaction with different B-subunits and also affect off-rates from any one subunit. Importantly, the B-subunit in the case of PR70 does not significantly reduce the A-subunit flexibility. For the entire trimeric complex, it is clear that the circular form of the A, B’’, and C subunits is constrained from the interaction between the B” and C subunits. Without the C subunit present, the A–B’’ subunit complex is similarly mobile as the A subunit by itself. The dynamic properties of the A-subunit are likely to be intrinsic to the dynamic events needed for the assembly of each of the different heterotrimeric PP2A complexes.

## Materials and Methods

### Gene construction

Multiple constructs (based on secondary structure prediction) of the genes coding for both isoforms of the A-subunit (*PPP2R1A* and *PPP2R1B*) as well as for different isoforms of the PR72/B-subunit (*PPP2R3A*, *PPP2R3B* and *PPP2R3C*) were cloned into the pNIC28-Bsa4 vector carrying a N-terminal Histidine-tag followed by a Tobacco Etch Virus (TEV) cleavage site using ligation independent cloning [41] and screened for expression and crystallization using established protocols [42], [43]. All expression vector vectors possess a T7 promoter and terminator sequence. All protein constructs were expressed in the *E. coli* strain BL21(DE3).

### Expression and purification

Typically cultures of 1L Terrific Broth (TB) medium in 2.5 L baffled conical flasks were inoculated from an overnight culture to a start OD_600 nm_ of 0.05 per ml and grown at 37°C at 200 rpm. Protein expression was induced at OD_600 nm_ of 0.7 with 0.2 mM IPTG and the temperature was lowered to 18°C. 16–20 hours after induction, cells were harvested at 10,000×g for 20 minutes and cells were resuspended in lysis buffer (1 g of cell wet weight in 5 ml lysis buffer; 20 mM Tris-HCl pH 7.5, 500 mM NaCl, 2 mM CaCl_2_, 5% glycerol, 0.5 mM TCEP, 1 EDTA-free Protease Inhibitor cocktail tablet/50 ml, 5 U/ml DNaseI) and incubated under stirring at 4°C for 60 minutes. Cells were disrupted with an Emulsiflex microfluidizer at 15,000 p.s.i. chamber pressure. Unbroken cells and cell debris were removed by centrifugation at 30,000 g for 30 min at 4°C. IMAC beads were incubated with the supernatant for 1 h and loaded in a plastic column (Biorad) and washed with wash buffer containing additional 10 mM and 20 mM imidazole, respectively. Purified protein was eluted with elution buffer containing 300 mM imidazole and subsequently dialysed against 20 mM Tris-HCl pH 7.5, 500 mM NaCl, 2 mM CaCl_2_, 5% glycerol, 0.5 mM TCEP together with 0.5 mg TEV protease overnight. Cleaved protein was passed over IMAC beads again and the flow through was collected, concentrated and subjected to gel filtration in 20 mM Tris-HCl pH 7.5, 200 mM NaCl, 5% glycerol, 2 mM CaCl_2_, 0.5 mM TCEP. For structure determination the PR70 (residues 130–483) construct was labeled with selenomethionine in M9 minimal media according to standard protocols.

### Limited proteolysis

To identify stable constructs for crystallization limited proteolysis was performed. PR70 and PR72 constructs at 0.4 mg/ml were incubated with chymotrypsin (ratio 100∶1) in a buffer of 20 mM Tris pH 7.5, 200 mM NaCl, 2 mM CaCl_2_ and 0.5 mM TCEP. The mix was incubated at RT for 360 minutes and aliquots were removed after 0, 5, 10, 15, 30, 60, 120, 240 and 360 minutes. Aliquots were mixed with SDS-PAGE sample buffer, heated and analyzed on a SDS-PAGE gel. Stable protein fragments were identified using mass spectrometry and new constructs designed.

### Crystallization and structure determination

The crystal of the PR70 core domain (residues 130–483) used for refinement was obtained by sitting drop vapor diffusion by mixing selenomethionine labeled protein at 6.3 mg/ml with reservoir solution (1∶2 ratio) containing 0.1 M Tris-HCL pH 7.,5 16% PEG 2000 monomethyl ether and 0.15 M trimethylamine n-oxide. Prior to data collection, the crystal was equilibrated in reservoir solution supplemented with 25% glycerol and flash frozen in liquid nitrogen. Data were collected at the selenium edge at Diamond Light Source beamline I04 and processed with XDS and XSCALE (Table 2) [44]. SAD phasing, density modification and initial model building was carried out using PHENIX autosol [45]. In total, 10 selenium sites were found representing all 9 methionine residues present in the construct (two closely situated sites were identified for Met-427). The model was completed by several iterations of manual rebuilding in Coot [46] and maximum likelihood refinement in PHENIX refine [47] using translation libration screw (TLS) restraints (Table 2). The final model was validated with MolProbity v4.02b [48]: Clash score was 1.79 (100^th^ percentile) and overall score was 0.94 (100^th^ percentile). Considering backbone geometry, 98.6% of the modeled residues were in favored regions in the Ramachandran plot and there were no outliers. For calculating RMSD values, we used either DALI; http://www.ebi.ac.uk/Tools/structure/dalilite/ [49] or PyMol (DeLano) [50]. PyMol was also used generating structure figures. Domain movements were analyzed using the DynDom server; http://fizz.cmp.uea.ac.uk/dyndom/ [51]. Coordinates and structure factors have been deposited in the PDB with the accession number 4MEW.

### Heat induced unfolding followed by Circular Dichroism spectroscopy

CD thermal unfolding of different B-subunit constructs was followed over a temperature range from 15–70°C at a heating rate of 1°C/min in 10 mM Tris pH 7.5, 100 mM NaCl, 50 µM EGTA with increasing concentrations of CaCl_2_. CD unfolding curves were recorded with a JASCO J600A spectropolarimeter (1 cm cell length, 1 µM protein concentration, 1 nm bandwidth) at 230 nm. Data were analyzed according to a two state model to obtain apparent unfolding midpoints using the software GraFit5. It should be noted that thermal unfolding was irreversible under all conditions measured.

### Isothermal titration calorimetry

ITC measurements were performed on an iTC200 instrument (GE Healthcare, Chalfont St. Giles, UK). Various B’’-constructs at a concentration of 20–40 µM in the calorimetric cell (total cell volume of 220 µl) were titrated with 200–300 µM of different Aα and Aβ constructs at 15°C. As buffer system, 20 mM Tris pH 7.5, 200 mM NaCl and 2 mM CaCl_2_ was used. The heat generated after each ligand injection was obtained by integration of the calorimetric signal. Resulting binding isotherms were analyzed according to a 1∶1 binding model using the Origin software (OriginLab Corp., Northampton, MA, USA).

### SAXS

SAXS data were collected at the SIBYLS 12.3.1 beamline at the Advanced Light Source, Lawrence Berkeley National Laboratory [52]–[54]. Scattering measurements were performed on 20–30 µl samples at 15°C, 1.5 meter from the Mar165 detector. A series of truncation constructs of the A- and PR70 subunits of protein phosphatase 2A individually and as complexes were purified on a Superdex 200 equilibrated in 20 mM Tris pH 7.5, 20 mM NaCl, 2 mM CaCl_2_, 0.5 mM TCEP, 1% glycerol at 15°C. Data were collected on the original gel filtration fractions from each SEC run, as well as on concentration series from fractions sampled from the later eluting half of each SEC elution peak (∼2–8-fold concentration). Fractions prior to the SEC void volume were used for buffer subtraction of the original unconcentrated gel filtration fractions, and concentrator eluates were used for buffer subtraction of each concentration series (2×−8×). Sequential exposures (0.5, 0.5, 2, 5, 0.5 seconds) were taken at 11.111 eV. Samples were radiation-sensitive, but for most samples, data from the first and second exposure measurements matched. For very radiation sensitive samples, the option “expose with mix” was applied. Here, 30 µl of sample were taken up in the Hamilton syringe and slowly pushed out during each exposure. No radiation-dependent aggregation was observed in sequential exposures. Data were analyzed using the ATSAS program suite [55], [56], SCATTER [52], [57], BilboMD [38], [58], [59], and the Volatility of Ratio server [39]. BilboMD models were generated with Rg allowed to range between 35 Å and 45 Å, 20 Å and 50 Å, 30 Å and 50 Å for heat15, complexes with B” subunit (25–575), complexes with B” subunit (130–575), respectively (table 5). BilboMD models were depicted using PyMOL [50].

**Table 5:** SAXS statistics.

	Conc. mg/ml	*Exp (s)*	q Range (Å^−1^)	*R_g_* Guinier	*R_g_ P(r)*	*D_max_*Å	*Exp. mass*	*Calc. mass*
A subunit	3.0	0.5	0.0125–0.316	37.5Å	37.4	120	63	65
A α +PR70 (130–575)	1.5	5	0.0137–0.323	41.9	42.4	145	112	117

## Supporting Information

Figure S1 **Sequence alignment of B″ regulatory subunits.** Secondary structural elements are indicated above the sequences. Conserved residues are highlighted and boxed in red. Residues that interact with the scaffolding subunit are highlighted with green circles. Residues that are involved in calcium binding are indicated by blue circles.
(TIF)

Figure S2 **Structural overlays between the CBLD domains and individual EF-lobes of PR70 and the top scoring hits from DALI.** All panels: PR70 is grey except for the calcium ions (yellow), and the DALI hits are blue except for calcium (orange). More information about the DALI hits can be found in Table 3. **A.** KChIP1 on PR70 CBLD1. **B.** CBL2 on PR70 CBLD2. **C.** Calmodulin on EF-lobe 1. **D.** Calcium binding protein p22 on EF-lobe 2. **E.** CDC31p on EF-lobe 3. **F.** KChiP1 on EF-lobe 4.
(TIF)

Figure S3 **Interaction interface between CBLD1 and CBLD2.** The interface encompasses helix E4 from CBLD1, helices E5 and F6 from CBLD2 and the inter-domain linker. E4, E5 and F6 are colored as in Fig. 2b (E4, yellow; E5, pale orange; F6, dark red), the linker is violet, the remainder of the protein is grey and the calcium ion at EF5 is yellow. Salt bridges and hydrogen bonds (length≤3.2 Å) are indicated by dashed lines and implicated residues are labeled. Nine such polar interactions were identified in the interface, though two are not visible in this view (between Arg260/Tyr-320 and between Tyr-261/main chain of Phe-311). In addition, several hydrophobic contacts and van der waal interactions can also be recognized. Note in particular that F6 from CBLD2 presents a large number of hydrophobic residues that pack against E5, also from CBLD2, and E4 from CBLD1.
(TIF)
