# Supplementary figures and images for: Structural and Biochemical Characterization of Human PR70 in Isolation and in Complex with the Scaffolding Subunit of Protein Phosphatase 2A
Source: PLoS One. 2014 Jul 9;9(7):e101846. doi: 10.1371/journal.pone.0101846 (PMC4090178; doi:10.1371/journal.pone.0101846)

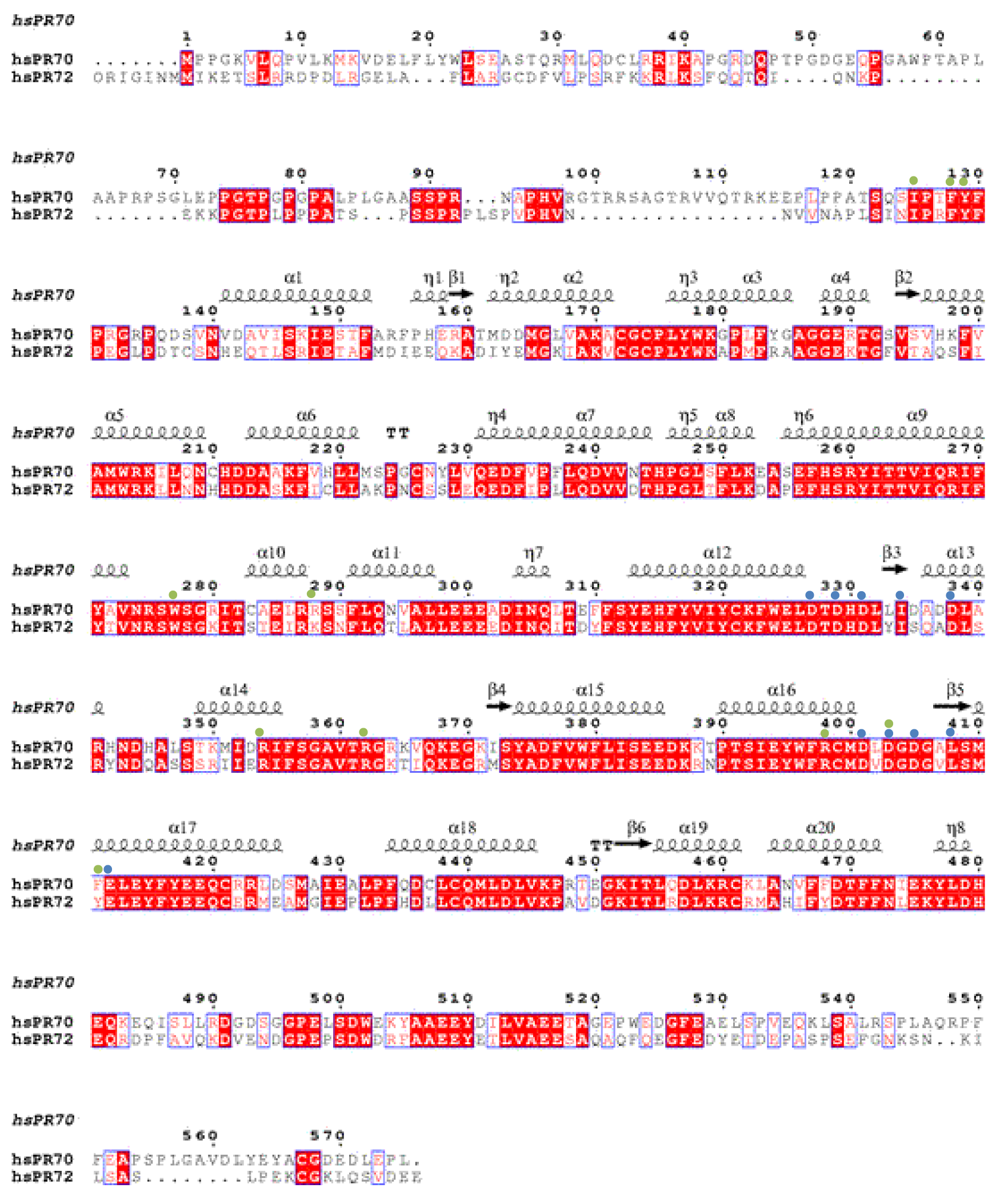

Supplement: Figure S1 — Sequence alignment of B″ regulatory subunits. Secondary structural elements are indicated above the sequences. Conserved residues are highlighted and boxed in red. Residues that interact with the scaffolding subunit are highlighted with green circles. Residues that are involved in calcium binding are indicated by blue circles. (TIF) [file pone.0101846.s001.tif]

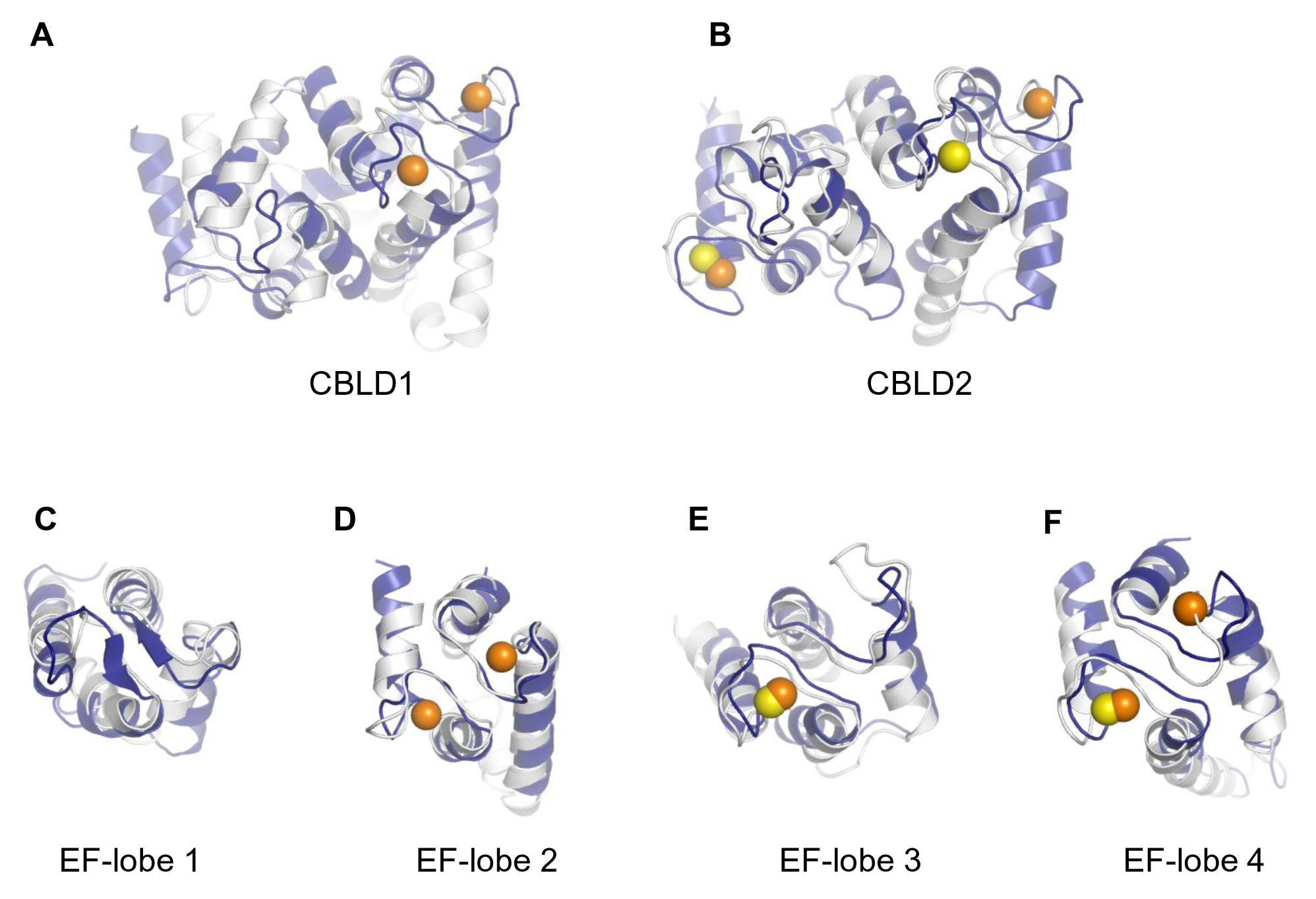

Supplement: Figure S2 — Structural overlays between the CBLD domains and individual EF-lobes of PR70 and the top scoring hits from DALI. All panels: PR70 is grey except for the calcium ions (yellow), and the DALI hits are blue except for calcium (orange). More information about the DALI hits can be found in Table 3. A. KChIP1 on PR70 CBLD1. B. CBL2 on PR70 CBLD2. C. Calmodulin on EF-lobe 1. D. Calcium binding protein p22 on EF-lobe 2. E. CDC31p on EF-lobe 3. F. KChiP1 on EF-lobe 4. (TIF) [file pone.0101846.s002.tif]

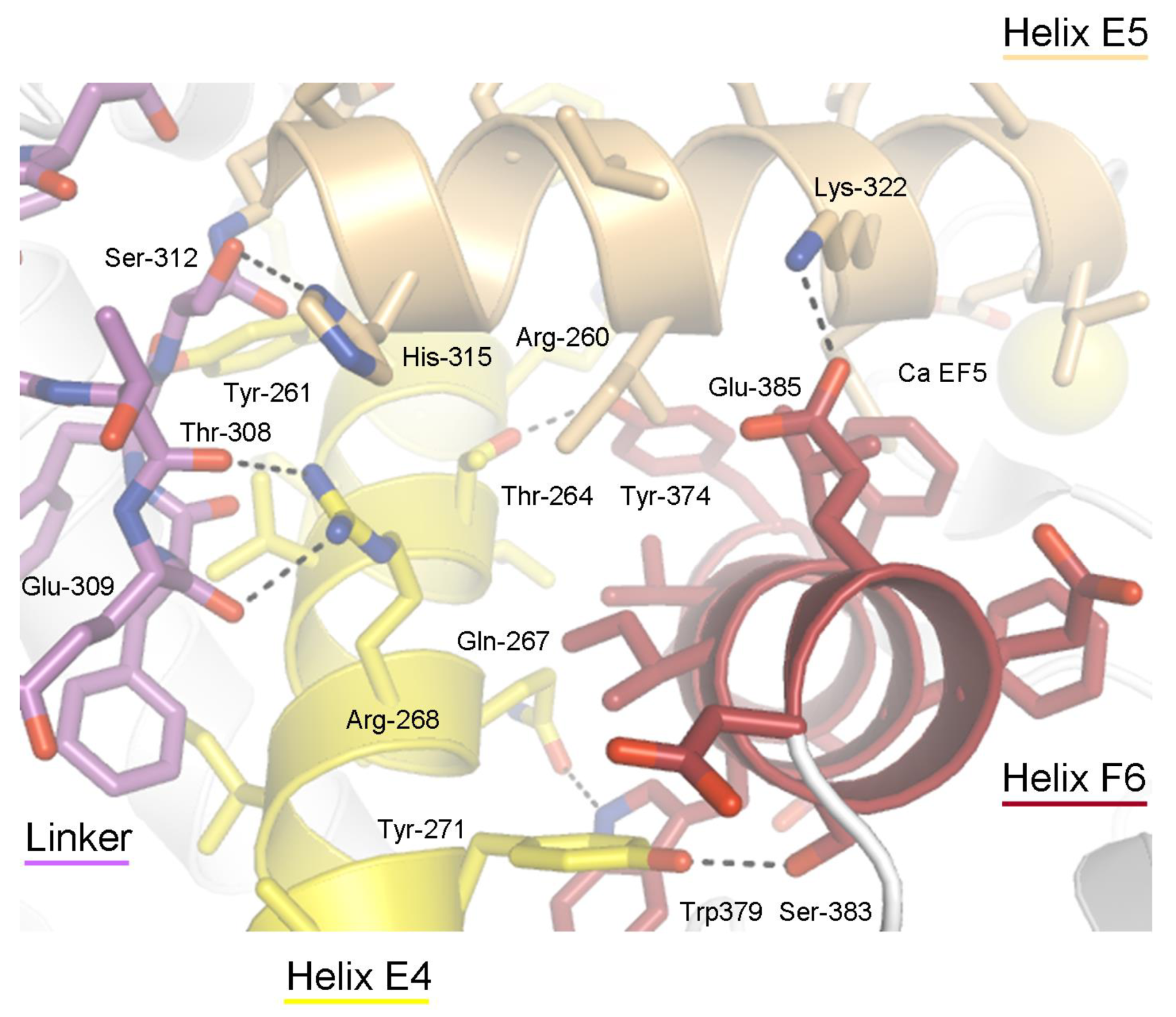

Supplement: Figure S3 — Interaction interface between CBLD1 and CBLD2. The interface encompasses helix E4 from CBLD1, helices E5 and F6 from CBLD2 and the inter-domain linker. E4, E5 and F6 are colored as in Fig. 2b (E4, yellow; E5, pale orange; F6, dark red), the linker is violet, the remainder of the protein is grey and the calcium ion at EF5 is yellow. Salt bridges and hydrogen bonds (length≤3.2 Å) are indicated by dashed lines and implicated residues are labeled. Nine such polar interactions were identified in the interface, though two are not visible in this view (between Arg260/Tyr-320 and between Tyr-261/main chain of Phe-311). In addition, several hydrophobic contacts and van der waal interactions can also be recognized. Note in particular that F6 from CBLD2 presents a large number of hydrophobic residues that pack against E5, also from CBLD2, and E4 from CBLD1. (TIF) [file pone.0101846.s003.tif]
